# Serotonin attenuates tumor necrosis factor-induced intestinal inflammation by interacting with human mucosal tissue

**DOI:** 10.1038/s12276-025-01397-1

**Published:** 2025-02-03

**Authors:** Veronika Bosáková, Ioanna Papatheodorou, Filip Kafka, Zuzana Tomášiková, Petros Kolovos, Marcela Hortová Kohoutková, Jan Frič

**Affiliations:** 1 International Clinical Research Center, St. Anne’s University Hospital, Brno, Czech Republic; 2 Department of Biology, Faculty of Medicine, Masaryk University, Brno, Czech Republic; 3 Department of Experimental Biology, Faculty of Science, Masaryk University, Brno, Czech Republic; 4 Department of Molecular Biology and Genetics, Democritus University of Thrace, Alexandroupolis, Greece; 5 International Clinical Research Center, Faculty of Medicine, Masaryk University, Brno, Czech Republic; 6 Institute of Hematology and Blood Transfusion, Prague, Czech Republic

**Keywords:** Monocytes and macrophages, Inflammation, Neuroimmunology

## Abstract

The intestine hosts the largest immune system and peripheral nervous system in the human body. The gut‒brain axis orchestrates communication between the central and enteric nervous systems, playing a pivotal role in regulating overall body function and intestinal homeostasis. Here, using a human three-dimensional in vitro culture model, we investigated the effects of serotonin, a neuromodulator produced in the gut, on immune cell and intestinal tissue interactions. Serotonin attenuated the tumor necrosis factor-induced proinflammatory response, mostly by affecting the expression of chemokines. Serotonin affected the phenotype and distribution of tissue-migrating monocytes, without direct contact with the cells, by remodeling the intestinal tissue. Collectively, our results show that serotonin plays a crucial role in communication among gut–brain axis components and regulates monocyte migration and plasticity, thereby contributing to gut homeostasis and the progression of inflammation. In vivo studies focused on the role of neuromodulators in gut inflammation have shown controversial results, highlighting the importance of human experimental models. Moreover, our results emphasize the importance of human health research in human cell-based models and suggest that the serotonin signaling pathway is a new therapeutic target for inflammatory bowel disease.

## Gut–brain axis insights reveal serotonin’s role in immunity

The gut–brain axis involves communication between the brain and the gut, which is important for maintaining gut health. Here the authors explored this by studying serotonin’s role in gut inflammation using a three-dimensional human cell model. They used intestinal organoids to mimic human gut conditions. These organoids were treated with serotonin and TNF to study their effects on gut cells and immune responses. The researchers found that serotonin reduced TNF-induced inflammation by altering gene expression related to immune cell movement. The study showed that serotonin can decrease the production of certain inflammatory signals in the gut, potentially reducing inflammation. This suggests that targeting serotonin could help treat inflammatory bowel disease (IBD). In conclusion, serotonin plays a role in controlling gut inflammation, offering insights into new treatments for IBD. Future research could explore serotonin’s broader impact on other inflammatory diseases.

This summary was initially drafted using artificial intelligence, then revised and fact-checked by the author.

## Introduction

The gut‒brain axis is defined as comprising bidirectional interactions between the central nervous system and the largest and most complex component of the peripheral nervous system—the enteric nervous system (ENS). As the gut mucosa is able to respond to neural signaling^1^ and intestinal enteroendocrine cells (EECs) represent a major source of some neuromodulators^2–7^, interactions between the intestinal tissue and the ENS are key mechanisms for maintaining gut homeostasis. How interactions between the intestinal tissue and the gut microbiome and the dysregulation of the mucosal immune system lead to an uncontrolled inflammatory response has been studied; however, the involvement of the ENS in these processes has not been well described.

The intestinal tissue harbors the largest reservoir of immune cells in the human body^8^; however, the interconnectivity between neuromodulators and the intestinal immune system has not been completely elucidated. Macrophages (Mφs) are abundant in intestinal tissue and exhibit remarkable functional plasticity in response to their microenvironment. Mφs contribute to maintaining tissue homeostasis but can exacerbate inflammation when dysregulated^9^. Intestinal Mφs mainly originate from embryo-derived precursors and have a self-maintenance ability^9–12^. However, the mature Mφ pool can also be replenished by circulating monocytes that migrate into the mucosal tissue^9,13^. In a pathological state, such as inflammatory bowel disease (IBD), monocyte–Mφ differentiation is dysregulated, leading to impaired bacterial clearance and the production of cytokines such as tumor necrosis factor (TNF)^14–18^, and exacerbating tissue damage. Therapeutic inhibition of TNF has revolutionized the management of IBD, suggesting that TNF plays a crucial role in the pathogenesis of intestinal inflammation. The role of TNF has been well studied in the context of inflammation, which it promotes by inducing cell death^19^. However, the effects of TNF stimulation on the mucosal tissue and its connection to the gut‒brain axis are still not fully understood because of the lack of suitable immune cell-depleted tissue and ENS models. Moreover, the development of MΦs differs between mice and humans, and studies are impeded by the difficulty in creating human cell models. Therefore, how the mucosal tissue influences monocyte migration, fate and differentiation in humans is still unclear.

Neuromodulators play crucial roles in regulating physiological functions and homeostasis in the human body, with the latter affecting multiple systems, including the nervous system, mucosal tissue and immune system. Various cells, including enteric neurons, immune cells, the gut microflora and intestinal EECs, are sources of neuromodulators. In particular, 50% of whole-body dopamine and more than 95% of total serotonin are produced in the gut, mainly by EECs^2–7^. In addition to their well-established roles in the nervous system, dopamine and serotonin exert pleiotropic effects on intestinal tissues^2^, and both are involved in the inflammation and pathogenesis of IBD^2,20^. The roles of serotonin in the development and progression of intestinal inflammation and related pathologies have been thoroughly studied and reviewed^20–23^. A variety of immune cells, including monocytes, express serotonin receptors and thus respond to serotonin stimulation^24,25^. In this context, serotonin stimulation is believed to promote monocyte chemotaxis toward sites of inflammation^26^; however, the precise mechanisms mediating these actions are unresolved.

Animal models have been widely used to investigate the mechanisms and pathogenesis of human inflammatory diseases. Although these models have generated important findings, rodents and humans differ in their susceptibility to pathogens and the microbiome composition. In mucosal tissue, Toll-like receptor (TLR) signaling is a vital part of innate immunity. Furthermore, given the ligand recognition specificity of each TLR, the expression and localization of the receptors are crucial for an adequate immune response. However, the expression of TLRs differs strikingly between mice and humans throughout the whole gastrointestinal tract^27^; therefore, the translation of in vivo results can be challenging. In the context of serotonin, controversial findings were reported when mouse and rat models were used to elucidate the role of this neuromodulator in intestinal inflammation^28–30^. Therefore, a human cell-based intestinal organoid (IO) model provides a solution for these conflicting results obtained with animal models. Organoids are three-dimensional (3D) ‘organ-in-a-dish’ models that aim to recreate key aspects of the in vivo structure of tissues using multiple cell types from the species of interest, generating a simulated microenvironment in which the organoid cells exhibit key aspects of organ function^31^. Previously, we showed that induced pluripotent stem (iPS) cell-derived IOs form an immunocompetent environment, facilitating the co-cultivation of immune cells and observations of their interactions with the mucosal tissue^32^. Therefore, IOs represent potent models for studying the roles of neuromodulators in homeostasis and the immune functions of the intestinal mucosal tissue.

Despite the accumulating evidence highlighting the crucial roles of neuromodulators, particularly serotonin, in intestinal pathophysiology, their underlying mechanisms of action are elusive. Here, we aimed to describe the effect of TNF stimulation on the mucosal tissue in a 3D human in vitro model. We hypothesized that serotonin is involved in the development and resolution of intestinal inflammation, affecting both the mucosal tissue and resident immune cells. Therefore, we aimed to describe the roles of serotonin in both homeostasis and TNF-induced inflammation using a human cell-based model, thus overcoming the limitations and interspecies differences associated with in vivo models. Current therapeutic strategies for IBD rely on attenuating intestinal inflammation using immunosuppressants, immunomodulators and biological treatments targeting specific cytokines or immune cell types. Despite the progress in the development of new agents, a notable portion of patients with IBD are primarily or secondarily nonresponsive to conventional treatment. Therefore, investigations of novel therapeutic approaches are emerging.

In this study, we elucidated the impacts of neuromodulators, including serotonin, on the expression profiles of human gut mucosal cells and infiltrating monocytes. We used state-of-the-art 3D in vitro organoid models depleted of all ENS and immune cells and ex vivo co-cultivation of IOs with human monocytes. For the first time, the direct effects of serotonin on the mucosal tissue during homeostasis and TNF-induced inflammation were studied. Our study sheds light on the mechanisms underlying the role of serotonin in human gut pathophysiology in the context of inflammation and provides clues to the bidirectional communication occurring in the gut‒brain axis. Taken together, our results suggest that therapeutics targeting serotonin might alleviate intestinal inflammation in patients with IBD.

## Materials and methods

### HiPS cell maintenance

Human iPS (hiPS) cells (WiCell, DF19-9-7T^33^) were cultured in Matrigel-coated tissue culture dishes and maintained in mTeSR Plus medium (StemCell Technologies) in an incubator at 37 °C with 5% CO_2_ and 95% humidity. The medium was changed every second day. When the cells reached ~80% confluence, they were passaged using TryPLE (Gibco) and exposed to a RHO/ROCK pathway inhibitor (10 μM; StemCell Technologies) for 24 h in an incubator at 37 °C with 5% CO_2_ and 95% humidity. The next day, the cells were washed with fresh medium and maintained until the next passage.

### Intestinal organoid differentiation

Human IOs were differentiated from hiPS cells using an adapted published protocol^32,34^. Briefly, iPS cells were induced to form 3D IOs via a three-step protocol. On day 0, hiPS cells were induced to undergo definitive endoderm differentiation using RPMI 1640 medium (Gibco) supplemented with activin A (100 ng/ml, R&D). The next day, the medium was changed, and fresh RPMI 1640 medium supplemented with activin A (100 ng/ml) and 0.2% HyClone-defined fetal bovine serum (FBS; GE Healthcare Bio-Sciences) was added. On the third and fourth days, the medium was changed, and RPMI 1640 medium supplemented with activin A (100 ng/ml) and 2% HyClone-defined FBS was added. The definitive endoderm was induced to undergo mid- and hindgut differentiation by changing the medium daily (RPMI 1640 medium supplemented with 15 mM HEPES, 2% HyClone-defined FBS, 500 ng/ml FGF4 (R&D) and 500 ng/ml WNT3a (R&D)) until 3D spheroids formed. Spheroids were collected, embedded in a drop of Cultrex Membrane Extract Type 2 (R&D) and fed IO complete medium (advanced Dulbecco’s modified Eagle medium F12 (Gibco) supplemented with B27 supplement (Thermo Fisher Scientific), GlutaMAX supplement (Thermo Fisher Scientific), penicillin and streptomycin (500 U/ml), 15 mM HEPES, 500 ng/ml R-spondin A (R&D), 100 ng/ml Noggin (R&D) and 100 ng/ml EGF (R&D)), and the medium was changed twice a week. After approximately 50 days, the IOs were used in the experiments.

### IF staining

IOs were fixed with 4% paraformaldehyde (PFA) for 20 min at room temperature (RT) and washed three times with PBS. For whole-mount staining, the IOs were permeabilized according to the following protocol. For histological slides, IOs were first dehydrated with 15% sucrose overnight at 4 °C. Next, the samples were frozen in tissue-freezing medium (Leica Biosystems) in isopropanol cooled to −80 °C and stored in a freezer at −80 °C. Next, the frozen samples were cut and rehydrated in PBS for 10 min. IO sections were permeabilized with PBS + 0.5% Triton-X100 for 15 min at RT and washed three times with immunofluorescence (IF) buffer (PBS + 0.2% Triton-X100 + 0.05% Tween-20). The samples were blocked with IF buffer + 2.5% bovine serum albumin (BSA) for 1 h at RT. The cells were labeled with primary antibodies (the dilutions and clones are listed in Supplementary Table 1) overnight at 4 °C, washed three times with IF buffer and incubated with secondary antibodies and phalloidin in IF buffer with 1% BSA for 2 h at RT. The IOs were washed three times with IF buffer and stained with 4,6-diamidino-2-phenylindole (DAPI) for 5 min at RT. Before imaging, the IOs were washed with IF buffer. IO sections were imaged with a Zeiss LSM 780 confocal microscope at 10× magnification. The images were processed using ImageJ software.

### Immunohistochemistry

The IOs were washed with PBS, fixed with 4% PFA for 20 min at RT and washed three times with PBS. The IOs were dehydrated with 15% sucrose overnight at 4 °C and frozen in tissue-freezing medium. The frozen samples were sectioned and the sections were rehydrated in PBS for 10 min. Endogenous peroxidases were blocked with 5% H_2_O_2_/methanol for 30 min at RT. The samples were washed with H_2_O and PBS and blocked with 1% BSA for 45 min at RT. The cells were labeled with primary antibodies (the dilutions and clones are listed in Supplementary Table 1) overnight at 4 °C, washed three times with PBS and incubated with biotinylated secondary antibodies (Supplementary Table 1) for 60 min at RT. The samples were washed, and the Vectastain ABC-HRP Kit (Vector Laboratories) and ImmPACT DAB Substrate kit (Vector Laboratories) were used to develop the signal. Next, the samples were counterstained with a hematoxylin solution for 5 min at RT, dehydrated with ethanol and mounted using Eukitt Quick-hardening mounting medium (Sigma-Aldrich). The samples were imaged using a Zeiss Axio Scan Z1 slide scanner.

### IO dissociation and flow cytometry analysis

The IOs were washed with cold Hank’s balanced salt solution to remove the cultrex and cut into small pieces with scissors. The cells were dissociated with TrypLE (Gibco) for 10 min at 37 °C with constant shaking. Every 5 min, the samples were pipetted to dissociate the cells completely. The suspension of cells was filtered through a 70 μm strainer and washed with cold Hank’s balanced salt solution + 2% FBS. The cells were centrifuged at 300*g* for 10 min at 4 °C and resuspended in staining buffer (PBS + 0.5% FBS + 2 mM EDTA). The cells were labeled with fluorochrome-conjugated antibodies for 30 min on ice (the dilutions and clones are listed in Supplementary Table 1), washed with staining buffer and injected into a SONY SA3800 spectral flow cytometer (Sony Biotechnology). The data were analyzed using FlowJo v11 software (BD Life Sciences).

### Stimulation of IOs

IOs older than 50 days were stimulated with IO medium containing chemical triggers but without growth factors. The effects of neuromodulators on intestinal tissue were studied using 10 µM serotonin hydrochloride (R&D), 10 µM dopamine hydrochloride (R&D), 10 µM noradrenaline bitartrate (R&D) and 10 µM acetylcholine chloride (R&D). TNF (10 ng/ml, R&D) was used to induce a proinflammatory milieu. For analyses using quantitative (q)PCR and RNA sequencing (RNAseq), IOs were stimulated for 4 h. For fluorescence staining and chemotaxis experiments, the organoids were stimulated for 24 h. The untreated controls were cultivated with fresh IO medium without triggers for the appropriate times.

### RNA extraction and gene expression

The cells were lysed with TRI Reagent (Merck), and RNA was isolated using an RNeasy Plus Micro kit (Qiagen) according to the manufacturer’s instructions. The isolated RNA was used for RNAseq, RT^2^ Profiler PCR Array or qPCR. Before the qPCR analysis, RNA was transcribed to cDNA using a high-capacity cDNA reverse transcription kit (Applied Biosystems) according to the manufacturer’s protocol. The qPCR analysis was performed with TaqMan TLR2, TLR3, TLR4, TLR5, TLR6, TLR7, TLR8 and TLR9 probes (details in Supplementary Table 2) and TaqMan Gene Expression Master Mix (Thermo Fisher Scientific) according to the manufacturer’s protocol. A StepOne Real-Time PCR System (Applied Biosystems) was used for the qPCR analysis.

### RT^2^ profiler PCR array

RNA was isolated from IOs according to the aforementioned protocol. cDNA was synthesized using the RT^2^ First Strand kit (Qiagen). The expression profiles were screened using the RT^2^ Profiler PCR Array Human Inflammatory Response and Autoimmunity (Qiagen) according to the manufacturer’s protocol. A LightCycler 480 Instrument II (Roche) was used for the qPCR analysis.

### RNAseq

The integrity of the isolated RNA (according to the aforementioned protocol) was measured with a fragment analyzer using an RNA Kit 15 nt (Agilent Technologies). Samples with an RNA integrity number ≥8 were used for sequencing. Then, 120 ng of total RNA was used as input for library preparation via the QuantSeq 3′ mRNA-Seq FWD with the UDI 12 nt Kit (v.2) (Lexogen) in combination with the UMI Second Strand Synthesis Module for the QuantSeq FWD. Quality control for the library quantity and size distribution was performed using a QuantiFluor dsDNA System (Promega) and the High Sensitivity NGS Fragment Analysis kit (Agilent Technologies). The final library pool was sequenced on a NovaSeq 6000 (Illumina) using the S4 Reagent kit v1.5 300 with cycles in pair-end mode, resulting in an average of 20 million reads per sample.

### RNAseq analysis

The raw reads were quality checked (FastQC) and preprocessed for adapter trimming (Trim Galore). The trimmed reads were then mapped (HISAT2 and SAMtools) to the human genome (genome version: Ensembl GRCh38), resulting in a total alignment rate of 45–55%. Only uniquely mapped reads were retained for downstream processing. The strandedness and counts of each gene were determined (HTseq). All downstream procedures were performed in the R v4.4.3 environment. The differential expression analysis was performed with the DESeq2 pipeline. The differential expression results were filtered for low counts, and genes were considered differentially expressed when they displayed a |log_2_(fold change)| ≥0.6 and a *P* value ≤0.05. Gene Ontology (GO) analysis and gene set enrichment analysis were performed with the package clusterProfiler and the desktop version of GSEA MSigDB, respectively. The results were visualized in the R v4.4.3 environment (ggplot2 and Complexheatmap).

### PBM cell isolation

Peripheral blood mononuclear (PBM) cells were isolated from fresh, healthy buffy coats from human blood samples (Department of Transfusion and Tissue Medicine of Brno University Hospital). Human whole-blood samples were diluted with 2% FBS in PBS before gradient centrifugation on Lymphoprep (StemCell Technologies). A percentage of the freshly isolated PBM cells were labeled with anti-human CD3, CD4, CD8, CD14, CD16, CD19 and CD45 antibodies (details in Supplementary Table 1), referred to as ‘baseline’ in the results. The remaining PMB cells were used for chemotaxis/migration assays.

### Monocyte isolation

Monocytes were isolated from healthy human donor buffy coats (Department of Transfusion and Tissue Medicine of Brno University Hospital) using the RosetteSep Human Monocyte Enrichment Cocktail (StemCell Technologies) according to the manufacturer’s protocol. Monocyte purity was assessed by flow cytometry using CD14, CD16, CD45 and HLA-DR antibodies (details in Supplementary Table 1), and dead cells were excluded using the LIVE/DEAD Fixable Green Dead Cell Stain kit (Invitrogen).

### Chemotaxis/migration assay

PBM cells isolated using the aforementioned protocol were used for chemotaxis/migration assays. The cells were seeded into the upper compartments of Transwell inserts (cellQUART, 5 µm pores) filled with advanced Dulbecco’s modified Eagle medium F12 supplemented with B27 supplement, GlutaMAX supplement, penicillin and streptomycin (500 U/ml) and 15 mM HEPES. The lower compartments were filled with supernatants from IOs treated with 10 ng/ml TNF, 10 µM serotonin hydrochloride or a combination of both for 24 h. After 2 h of incubation, the cells that had migrated to the lower compartment were collected, washed and labeled with anti-human CD3, CD4, CD8, CD14, CD16, CD19, and CD45 antibodies (details in Supplementary Table 1). Dead cells were excluded using the LIVE/DEAD Fixable Violet Dead Cell Stain kit (Invitrogen). The cells were analyzed using a SONY SA3800 spectral flow cytometer (Sony Biotechnology).

### Co-cultivation of IOs and monocytes

First, IOs were stimulated with 10 ng/ml TNF, 10 µM serotonin hydrochloride or a combination of both for 24 h and then extensively washed with cold PBS. Monocytes isolated via the aforementioned protocol were mixed with IOs (10^5^ monocytes per organoid) by seeding them in a drop of cultrex. After 6 h, the organoids were extensively washed with cold PBS and dissociated according to the aforementioned protocol. The single cells in suspension were labeled with anti-human CD14, CD16, CD36, CD45, CD86 and HLA-DR antibodies (details in Supplementary Table 1). Dead cells were excluded using 7-AAD viability staining solution (eBioscience). The cells were analyzed using a SONY SA3800 spectral flow cytometer. The data were analyzed using FlowJo v11 software. For IF staining, monocytes and IOs were co-cultivated for 24 h, and after extensive washing, they were fixed with 4% PFA for 20 min at RT. The fixed samples were used for histological slide preparation according to the protocol described above. The samples were stained with anti-human CD45, CD90 and E-cadherin antibodies (details in Supplementary Table 1).

### Statistical analyses

Statistical analyses were performed with GraphPad Prism 9, unless indicated otherwise. The results are shown as the means and s.e.m. The data were tested for normality before parametric or nonparametric statistical tests were applied.

## Results

### Organoids form a complex environment for studying mucosal homeostasis

**Fig. 1:**
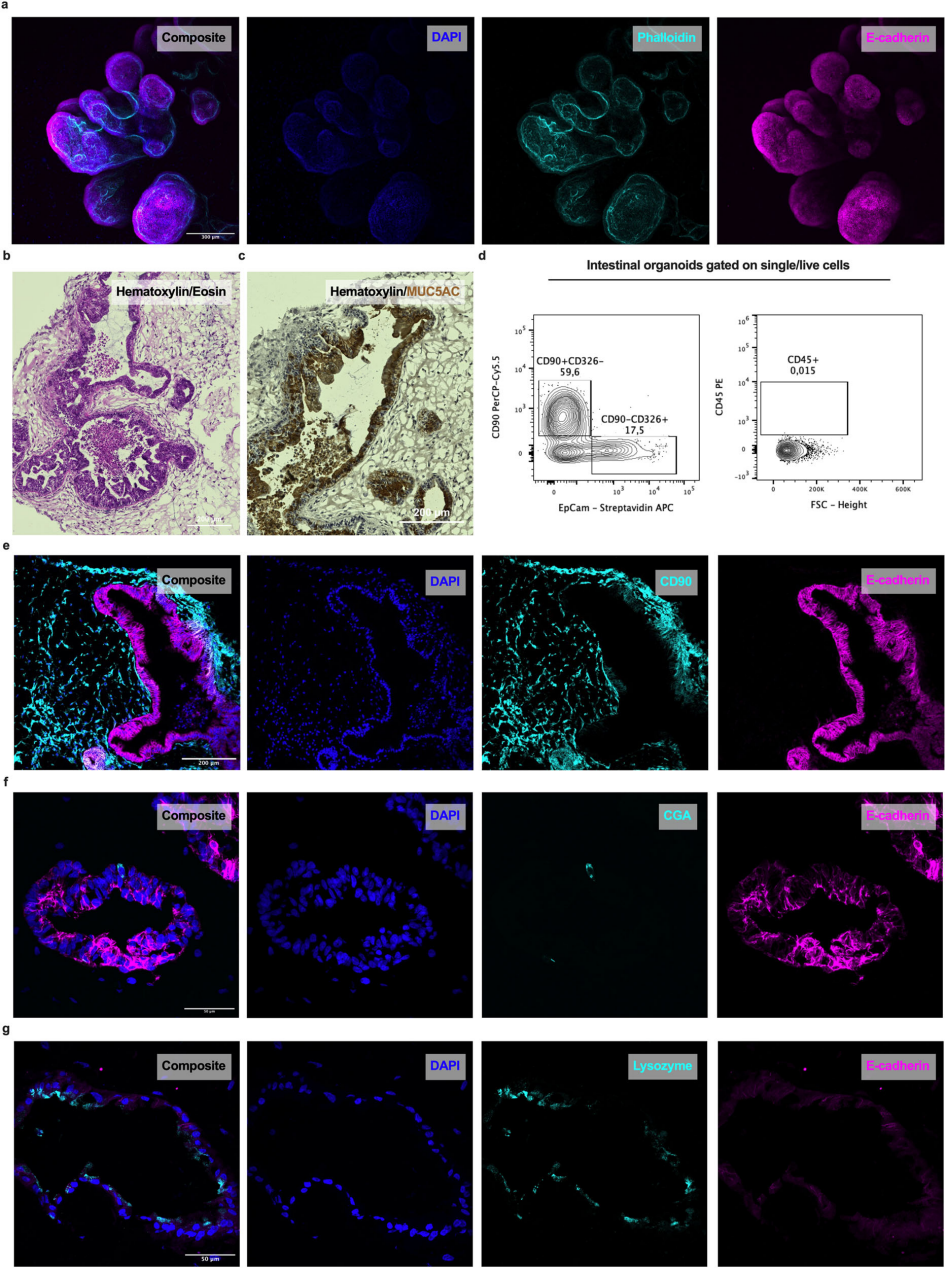
iPS cell-derived organoids form complex organized structures containing enteroendocrine and Paneth cells. **a** Whole-mount IF labeling for E-cadherin (magenta) and phalloidin (cyan) was performed, and the samples were counterstained with DAPI (blue), which depicts the polarity of the IOs. Scale bar, 300 µm. **b** Hematoxylin and eosin staining showing the structural organization of IOs. Scale bar, 200 µm. **c** Immunohistochemical labeling for MUC5AC in IOs counterstained with hematoxylin. Scale bar, 200 µm. **d** Flow cytometry analysis revealing the expression of CD90, EpCam and CD45 in IO cells. **e**‒**g** IF labeling for **e** E-cadherin (magenta) and CD90 (cyan), scale bar, 200 µm; **f** E-cadherin (magenta) and CGA (cyan), scale bar, 50 µm; and **g** E-cadherin (magenta) and lysozyme (cyan), scale bar, 50 µm; and counterstaining with DAPI (blue), showing epithelial, mesenchymal, enteroendocrine and Paneth cells within IOs.

The mucosal tissue consists of epithelial cells that abundantly express E-cadherin and form a polarized layer, with a brush border enriched in F-actin facing the lumen of the gut. In this study, we used hiPS cell-derived IOs to simulate the mucosal tissue. First, we mapped the complex structures of the IOs by performing IF labeling for F-actin (phalloidin) and E-cadherin (Fig. 1a). We investigated the polarization of the tissue using hematoxylin and eosin staining (Fig. 1b) and visualized the presence of organized structures, with the inner/lumen side of the organoid facing the lumen. Immunohistochemical labeling revealed the expression of MUC5AC by epithelial cells (Fig. 1c), indicating the mucus-producing functionality of the IO tissue. Next, flow cytometry analysis was employed to determine the presence of epithelial (CD90^−^EpCam^+^) cells and mesenchymal (CD90^+^EpCam^−^) cells (Fig. 1d, left), as well as the absence of immune cells, as evidenced by the lack of CD45^+^ cells (Fig. 1d, right). Using IF labeling, we further verified that the epithelial and mesenchymal cells had formed organized structures within the IOs (Fig. 1e), allowing us to study the homeostasis of the epithelial barrier. The results revealed that the IOs contained cells expressing chromogranin A (CGA), a marker of EECs (Fig. 1f), and lysozyme, a marker of Paneth cells (Fig. 1g), indicating the relevance of each IO as a model for studying the gut‒brain axis. Taken together, the results demonstrated that the IOs resembled human intestinal tissue in vivo in terms of their structure and functionality, facilitating our study of the mechanisms of homeostasis maintenance and communication within the gut‒brain axis in healthy mucosal tissue.

### TNF stimulation of IOs initiates chemotaxis and a proinflammatory cytokine response in intestinal tissue

**Fig. 2:**
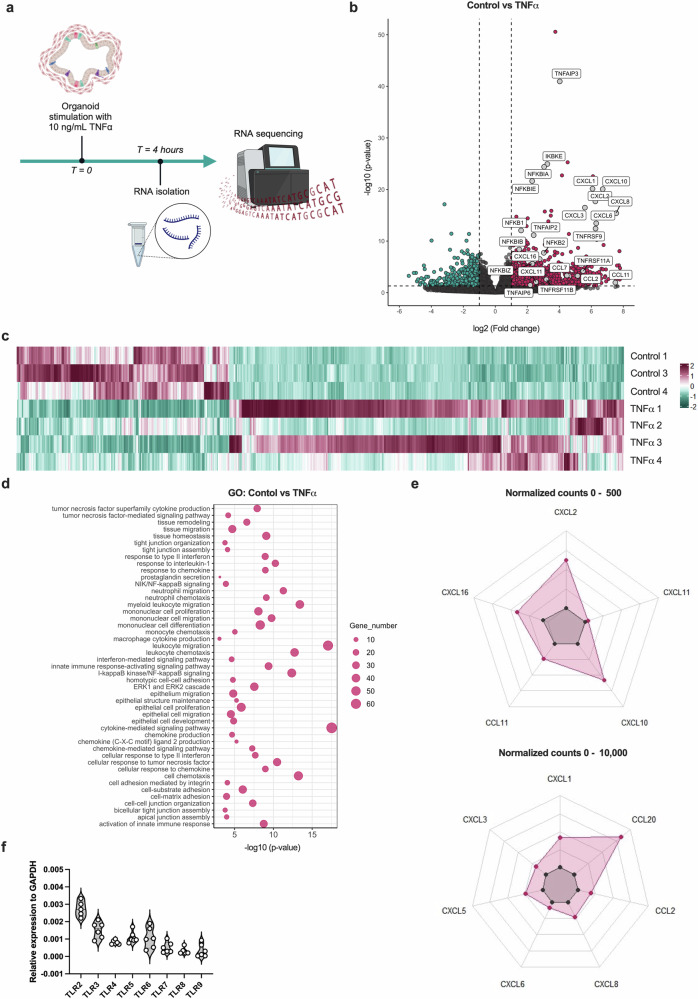
TNF induces a proinflammatory environment in organoid tissues. **a**, A scheme of in vitro experiments in which the IOs was stimulated with TNF for 4 h and changes in gene expression were analyzed using RNAseq. **b**, A volcano plot depicting DEGs in the TNF-treated samples compared with the untreated control samples. Magenta and cyan dots represent DEGs with *P* < 0.05. The dashed vertical lines represent a |log_2_(fold change)| of 1, and the horizontal dashed line represents a *P* value of 0.05. **c**, A heat map showing the expression of DEGs in the TNF-treated samples compared with the untreated control samples using scaled normalized counts. On the scale, magenta represents the highest expression and cyan represents the lowest expression of the genes. **d**, A bubble chart depicting the most significantly enriched pathways identified in the GO analysis. The bubble size corresponds to the number of genes involved in the corresponding pathway and the *x* axis shows significance based on the log_10_ value (*P* value). **e**, Spider plots showing the expression of genes as the mean of normalized counts, between 0 and 500 and between 0 and 10,000. The expression of chemokine genes among the significant DEGs is shown. **f**, A violin plot depicting TLR RNA expression in IOs. The data are presented as individual values along with the approximate frequency of data points shown as the width of each curve. The dashed lines represent the medians, and the dotted lines represent the first and third quartiles, *N* = 6.

Next, TNF, one of the main drivers of inflammatory responses, was used to stimulate the IOs to define their capacity to model the progression of inflammation in mucosal tissue. IOs were treated with recombinant human (rh) TNF for 4 h and, subsequently, global transcriptomic alterations were assessed via bulk RNAseq (Fig. 2a). The differential expression analysis of the RNAseq data revealed 1,415 differentially expressed genes (DEGs), of which 496 (35%) were downregulated and 919 (65%) were upregulated in the TNF-treated IOs compared with the untreated controls (Fig. 2b,c). GO analysis revealed several enriched pathways relevant to the response of IOs to TNF stimulation, including TNF-mediated signaling and the I-κB kinase/NF-κB signaling pathways (Fig. 2d). Interestingly, pathways associated with the chemotaxis of immune cells, such as leukocyte chemotaxis, monocyte chemotaxis, neutrophil chemotaxis and chemokine production (Fig. 2d)*,* were also enriched in the IOs treated with TNF, with the most abundant DEGs in these pathways being CCL2, CCL11, CCL20, CXCL2 and CXCL8 (Fig. 2e). These results illustrate the important role of mucosal cells in shaping the progression of inflammation, which they achieve via the modulation of immune cell chemoattractant signals. TLR signaling is an important initiator of the inflammatory response, inducing the expression of proinflammatory cytokines and chemokines to attract immune cells to the site of inflammation^35^. Thus, we sought to investigate the expression of TLRs in IOs. We observed high expression of TLR2, TLR3, TLR4, TLR5 and TLR6 (Fig. 2f), as expected for human intestinal tissue. The above findings demonstrate that IOs represent relevant models to facilitate our study of intestinal inflammation and the importance of TLR signaling and subsequent alterations in chemotaxis in mucosal cell inflammatory responses. Thus, we hypothesized that TLR signaling and downstream processes in the mucosal layer might be controlled by neuromodulators.

### Neuromodulators regulate TLR expression in IOs

**Fig. 3:**
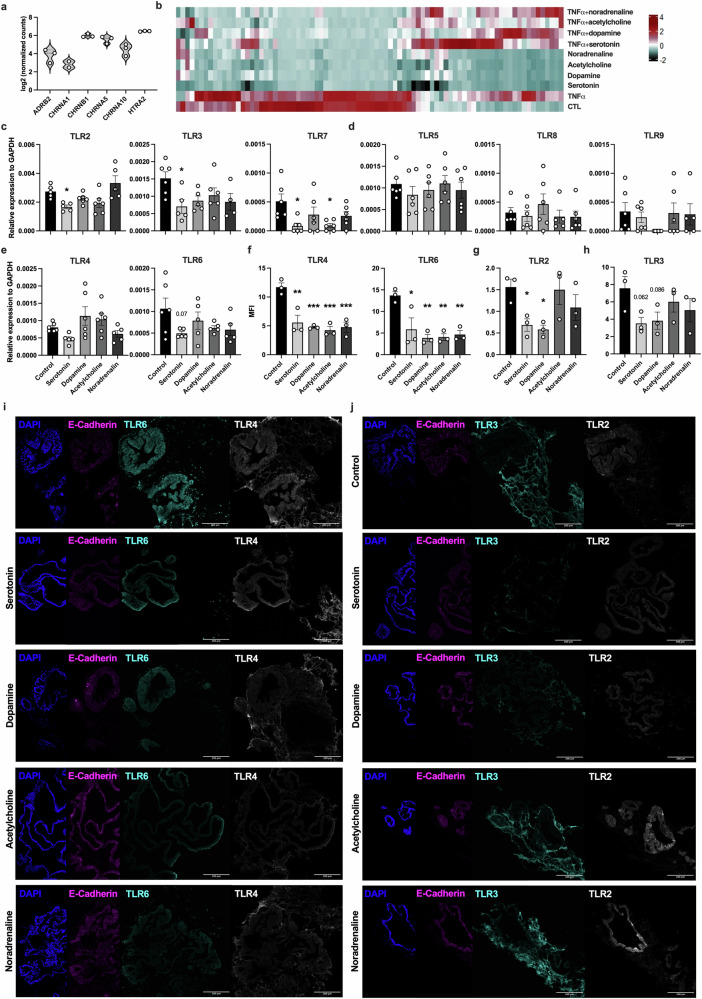
Neuromodulators regulate TLR signaling in the intestinal tissue. **a**, A violin plot depicting the RNA expression of neuromodulator receptors in IOs. The data are presented as individual values along with the approximate frequency of data points shown as the width of each curve. The dashed lines represent the medians and the dotted lines represent the first and third quartiles, *N* = 3. **b**, A heat map showing the results from the RT^2^ Profiler PCR array (human inflammatory response and autoimmunity) to screen for gene expression in IOs after serotonin, dopamine, acetylcholine and noradrenaline stimulation. On the scale, magenta represents the highest expression and cyan represents the lowest expression of the genes. **c**–**e**, mRNA expression of TLR2, TLR3 and TLR7 (**c**); TLR5, TLR8 and TLR9 (**d**); and TLR4 and TLR6 (**e**) in IOs treated with the indicated neuromodulators. The data are shown as individual values along the means ± s.e.m, *N* = 4–6. **f**–**h**, Quantification of the mean fluorescence intensity of IF labeling for TLR4 and TLR6 (**f**), TLR2 (**g**) and TLR3 (**h**) in IOs stimulated with the indicated neuromodulators. The data are shown as individual values along with the means ± s.e.m., *N* = 3. **i**, **j**, Representative images of IF labeling for TLR4 and TLR6 (**i**) and TLR2 and TLR3 (**j**), and the epithelial cell marker E-cadherin in IOs that were untreated or treated with serotonin, dopamine, noradrenaline or acetylcholine and counterstained with DAPI (blue). Scale bar, 200 µm; *N* = 3. Statistical significance was determined by ordinary one-way analysis of variance (ANOVA), *P < 0.05, **P < 0.01 and ***P < 0.005.

Considering these findings, we sought to determine the potential effects of neuromodulators on TLR signaling and the subsequent proinflammatory processes in the mucosal tissue. First, with our RNAseq data, we ascertained that the IOs expressed the neuromodulator receptors ADRB2, CHRNA1, CHRNB1, CHRNA5, CHRNA10 and HTRA2 (Fig. 3a). Next, we sought to determine whether relevant neuromodulators alter TLR signaling or stimulate the inflammatory response in mucosal IOs under physiological conditions. For this purpose, we stimulated the organoids with the neuromodulators serotonin, dopamine, noradrenaline and acetylcholine. First, we used an RT^2^ Profiler PCR array (human inflammatory response and autoimmunity) to screen the responses to the mentioned neuromodulators (Fig. 3b). Interestingly, the results indicated that the expression of various TLRs was decreased in the presence of different neuromodulators. We corroborated this finding by determining the RNA expression levels of TLRs in IOs that were untreated or treated with serotonin, dopamine, noradrenaline or acetylcholine using qPCR. After stimulation with serotonin, the IOs showed significantly decreased expression of TLR2, TLR3 and TLR7 (Fig. 3c), no change in the expression of TLR5, TLR8 and TLR9 (Fig. 3d), and a trend toward decreased TLR4 and TLR6 (Fig. 3e) expression. We also observed a significant decrease in TLR7 (Fig. 3c) expression after acetylcholine stimulation. We confirmed the above findings at the protein level by performing IF staining of IOs on histological slides (Fig. 3f–j), with a focus on TLR2, TLR3, TLR4 and TLR6, which were the most highly expressed TLRs in IOs (Fig. 2f). Quantification of the IF results revealed a significant decrease in TLR4 and TLR6 expression in all treatment groups compared with that in the untreated controls (Fig. 3f). We also observed a significant decrease in TLR2 expression (Fig. 3g) and a trend toward decreased TLR3 expression after serotonin and dopamine treatment (Fig. 3h). Given the abundance of TLR2, TLR3, TLR4 and TLR6, as shown in Fig. 2f, we concluded that neuromodulators governed important immune response pathways in the IOs and thus in vivo mucosal tissue by modulating the expression of TLRs.

### Serotonin exerts a modest non-proinflammatory effect on healthy mucosal tissue

**Fig. 4:**
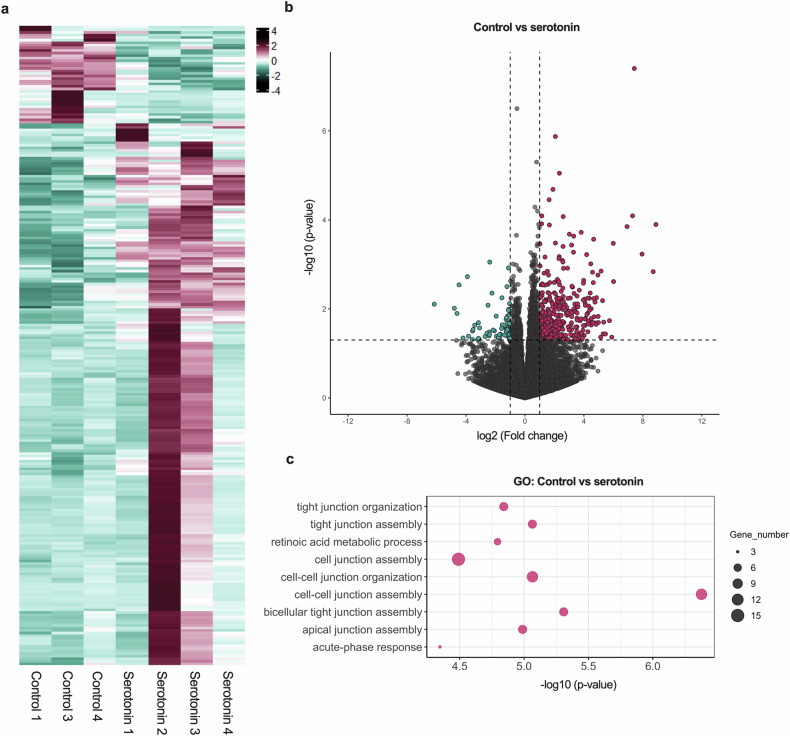
Serotonin has a mild effect on healthy intestinal tissue. **a**, A heat map showing the expression of DEGs in serotonin-treated samples compared with untreated controls based on scaled normalized counts. On the scale, magenta represents the highest expression and cyan represents the lowest expression of the genes. **b**, A volcano plot depicting DEGs in serotonin-treated IOs compared with untreated IOs. Magenta and cyan dots represent DEGs at *P* < 0.05. The dashed vertical lines represent a |log_2_ (fold change)| of 1, and the dashed horizontal line represents *P* = 0.05. **c**, A bubble chart showing the most significantly enriched pathways in serotonin-treated IOs compared with untreated controls obtained identified using GO analysis. The bubble size corresponds to the number of genes involved in the corresponding pathway and the *x* axis shows significance based on the log_10_ value (*P* value).

Next, we aimed to explore more deeply the effects of neuromodulators on intestinal tissue homeostasis using our RNAseq dataset. As we observed that serotonin had the most prominent effect on the IOs, we decided to focus only on stimulation with serotonin. First, we stimulated IOs with serotonin and compared them with untreated IOs as a control to test the effect of serotonin on healthy mucosal tissue. Serotonin influenced healthy tissue by altering its gene expression pattern (Fig. 4a). However, the analysis revealed only 249 DEGs in our dataset, of which 211 (85%) were upregulated and 38 (15%) were downregulated after serotonin treatment compared with the untreated control (Fig. 4a,b), suggesting that serotonin did in fact affect the tissue under physiological conditions. The GO analysis of these DEGs revealed that the enriched pathways in the serotonin-treated samples were related to tissue remodeling and included tight junction assembly and cell‒cell junction assembly (Fig. 4c). Therefore, in healthy intestinal tissue, serotonin has a subtle effect, inducing the remodeling of epithelial barrier integrity through tight junction assembly.

### Serotonin decreases chemokine expression in TNF-induced intestinal inflammation

**Fig. 5:**
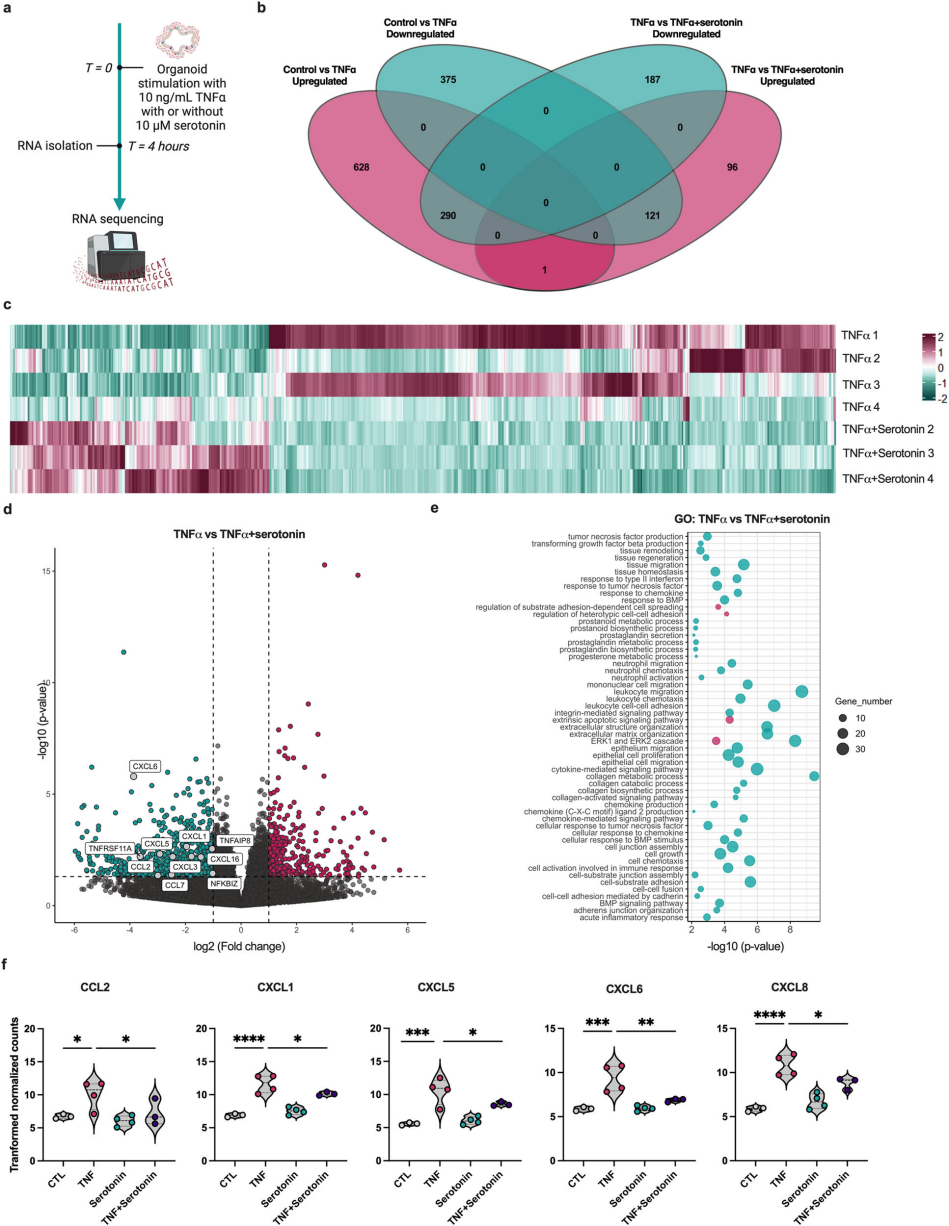
Serotonin affects the expression of chemokines in the proinflammatory state of the intestinal tissue. **a**, A schematic of in vitro experiments using IOs. IOs were stimulated with TNF in the presence or absence of serotonin for 4 h, and changes in total expression were assessed using bulk RNAseq. **b**, A Venn diagram depicting the numbers of common and unique DEGs identified in the TNF- and TNF + serotonin-treated samples compared with the TNF-treated and untreated controls. Magenta refers to the upregulated genes and cyan refers to the downregulated genes. **c**, A heat map showing the expression of DEGs in the serotonin-treated samples compared with the TNF + serotonin-treated samples, as determined using scaled normalized counts. On the scale, magenta represents the highest expression and cyan represents the lowest expression of the genes. **d**, A volcano plot depicting DEGs identified in TNF-treated IOs compared with TNF + serotonin-treated IOs. Magenta and cyan dots represent DEGs with *P* < 0.05. The dashed vertical lines represent |log_2_(fold changes)| of 1 and the horizontal dashed line represents *P* = 0.05. **e**, A bubble chart pinpointing the most significantly enriched pathways obtained using GO analysis. Magenta represents upregulated pathways, and cyan represents downregulated pathways. The bubble size corresponds to the number of genes involved in the corresponding pathway and the *x* axis shows significance based on the log_10_ value (*P* value). **f**, Violin plots showing the expression of CCL2, CXCL1, CXCL5, CXCL6 and CXCL8 in control (CTL) and TNF-, serotonin- and TNF + serotonin-treated samples. The data are presented as individual values along with the approximate frequency of data points shown as the width of each curve. The dashed lines represent the medians, and the dotted lines represent the first and third quartiles, *N* = 3–4. Statistical significance was determined by ordinary one-way ANOVA, *P < 0.05, **P < 0.01, ***P < 0.005 and ****P < 0.001.

Thus far, our findings support the idea that serotonin has a modest effect on the intestinal mucosal tissue under physiological conditions. We also showed that serotonin, along with other neuromodulators, affects the immune response by altering TLR expression in the mucosal tissue. Interestingly, previous research has also shown the possible interplay between the serotonin and TNF signaling pathways^30,36^. Thus, we aimed to describe the role of serotonin in inflammation by taking advantage of both our IO model of TNF-induced inflammation and our RNAseq dataset (Fig. 5a). In the presence of serotonin, we observed many fewer DEGs (695) than in the IOs treated with TNF alone (1,415). Furthermore, while the majority of genes were upregulated (919 (65%)) in TNF-treated organoids, in the presence of both TNF and serotonin, most genes were downregulated (477 (69%)), suggesting that serotonin attenuated the effects of TNF stimulation. We determined the common and unique gene expression patterns between these two conditions to confirm this hypothesis (Fig. 5b). Strikingly, we observed that more than 30% of the genes that were upregulated in the TNF-treated samples were downregulated in the presence of serotonin, and more than 20% of the downregulated genes in the TNF-treated samples were upregulated in the presence of serotonin, suggesting that serotonin attenuated TNF-induced inflammation. Next, we performed a detailed analysis of the dataset, comparing TNF + serotonin-treated IOs and TNF-treated IOs. The presence of serotonin induced a consistent response, as shown in the heat map (Fig. 5c). Most of the DEGs in this comparison were downregulated (Fig. 5d). We performed a GO analysis to determine whether the genes that were differentially expressed in the presence of serotonin participate in important pathways. The gene functions were enriched mainly in pathways involved in the chemotaxis and migration of leukocytes (Fig. 5e), suggesting that serotonin potentially attenuates the course of TNF-induced inflammation by influencing the movement of immune cells. Therefore, we focused on the main chemokines induced by TNF and the changes in their expression in the presence of serotonin. We detected a significant decrease in the expression of CCL2, CXCL1, CXCL5, CXCL6 and CXCL8 (Fig. 5f) in the TNF + serotonin-treated IOs compared with the TNF-only group. Notably, none of these chemokines were affected by serotonin stimulation in healthy tissue (Fig. 5f).

### Serotonin alters the ability of the intestinal tissue to attract monocytes during TNF-induced inflammation

**Fig. 6:**
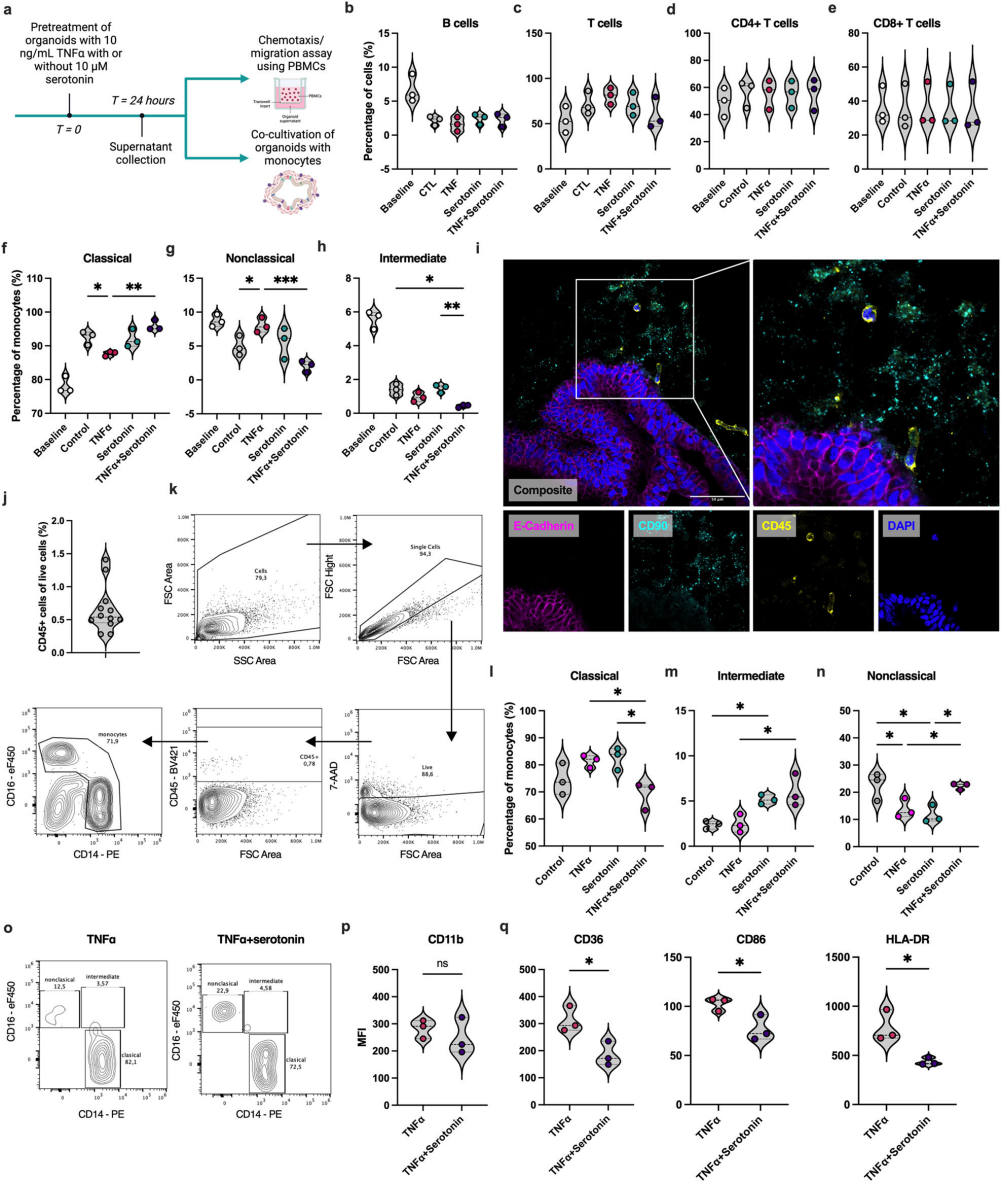
Serotonin influences the chemotaxis and fate of monocytes in the intestinal tissue under proinflammatory conditions. **a**, A schematic of in vitro experiments focused on the chemotaxis and migration of PBMCs and monocytes. IOs were treated with TNF in the presence or absence of serotonin, and after 24 h the supernatants were collected and used for chemotaxis assays with Transwell inserts. Stimulated organoids were used for co-cultivation with monocytes. **b**–**e**, The percentages of B cells (**b**), T cells (**c**) and CD4^+^ (**d**) and CD8^+^ (**e**) T cells among live cells that were chemoattracted and migrated toward the supernatants of IOs that were untreated (CTL) or treated with TNF, serotonin or TNF + serotonin analyzed using flow cytometry. **f**–**h,** The percentages of classical (**f**), nonclassical (**g**) and intermediate (**h**) monocytes that were chemoattracted and migrated toward the supernatants of IOs that were untreated (control) or treated with TNF, serotonin or TNF + serotonin analyzed using flow cytometry, *N* = 3. For plots in (**b**–**h**), the baseline represents the percentages of the respective cell types among the total PBMCs labeled immediately after isolation. **i**, IF labeling for E-cadherin, CD90 and CD45 in IOs co-cultivated with monocytes, displaying their migration toward IO tissue that was counterstained with DAPI (blue). Scale bar, 50 µm. **j**, the percentages of live CD45^+^ cells determined via a flow cytometry analysis of dissociated IO cells co-cultivated with monocytes. **k**, Representative contour plots of the flow cytometry gating strategy for monocytes co-cultivated with IOs. **l**‒**n**, The percentages of classical (**l**), nonclassical (**m**) and intermediate (**n**) monocytes cultivated with IOs stimulated with TNF serotonin or TNF + serotonin analyzed using flow cytometry, *N* = 3. **o**, Representative contour plots showing the expression of CD16 and CD14 by monocytes co-cultivated with IOs pretreated with TNF with or without serotonin, as analyzed using flow cytometry. **p**, **q**, The expression of CD11b (**p**), CD36, CD86 and HLA-DR (**q**) by monocytes co-cultivated with IOs stimulated with TNF in the presence or absence of serotonin, *N* = 3. For violin plots, the data are presented as individual values along with the approximate frequency of data points shown as the width of each curve. The dashed lines represent the medians and the dotted lines represent the first and third quartiles. Statistical significance was determined by ordinary one-way ANOVA (in **b**–**e**, **f**–**h** and **l**–**m**) and unpaired *t*-tests (**p** and **q**), *P < 0.05, **P < 0.01, ***P < 0.005 and ns, not significant.

We performed chemotaxis and migration assays to determine whether the serotonin-induced changes in inflammatory cell pathway-related genes translate to functional effects (Fig. 6a). We studied how the chemokines released by IOs affect the migration of peripheral blood mononuclear cells (PBMCs) by seeding PBMCs from healthy donors into Transwell inserts and incubating them with the supernatant from IOs treated with TNF in the presence or absence of serotonin (Fig. 6a, top). A flow cytometry analysis was performed to determine the effects of serotonin on the chemoattraction of different subpopulations of immune cells. The results revealed no differences in the chemoattraction of B cells (Fig. 6b), total T cells (Fig. 6c), CD4^+^ T cells (Fig. 6d) or CD8^+^ T cells (Fig. 6e) among the treatment groups. However, we observed significantly decreased chemoattraction of classical (CD14^+^CD16^−^) monocytes (Fig. 6f) and increased chemoattraction of nonclassical (CD14^−^CD16^+^) (Fig. 6g) monocytes in the presence of the supernatant from TNF-treated IOs. The chemoattraction of intermediate (CD14^+^CD16^+^) monocytes was not affected by TNF treatment (Fig. 6h). In agreement with our previous results (Fig. 5f), serotonin treatment of healthy tissue had no effect on the chemoattraction of any of the abovementioned cell types (Fig. 6b–h). However, when the supernatant from IOs treated with both TNF and serotonin was used, we observed a significant increase in classical monocyte chemoattraction (Fig. 6f) and a decrease in nonclassical (Fig. 6g) monocyte chemoattraction compared with the observations with the TNF only-treated IO supernatant. We also observed a significant decrease in the chemoattraction of intermediate monocytes when the supernatant from the TNF + serotonin-treated samples was used compared with the supernatant from the serotonin-treated or untreated samples (Fig. 6h).

### Serotonin remodels the mucosal tissue to affect the phenotype and fate of tissue-migrating monocytes

Given the effects of serotonin on the chemoattraction and migration of monocytes, the remodeling of healthy tissue, and the attenuation of inflammatory signaling pathways, such as TLR signaling, obtained in the previous steps, we sought to determine whether serotonin alters monocyte migration by remodeling IO tissue. Our group has previously shown that IOs can be co-cultured and interact with human monocytes^32^. Therefore, we conducted co-cultivation of monocytes isolated from healthy donors with IOs pretreated with TNF with or without serotonin (Fig. 6a, bottom). First, we assessed the presence of monocytes migrating toward IO tissue by preparing histological slides and performing IF staining. After 24 h of co-cultivation, we were able to detect CD45^+^ cells within the IO tissue in close proximity to mesenchymal and epithelial cells (Fig. 6i). The flow cytometry analysis of dissociated IOs co-cultivated with monocytes revealed the presence of CD45^+^ cells (0.6255 ± 0.3514%) (Fig. 6j), which were further identified as monocytes based on the expression of the CD14 and CD16 markers (Fig. 6k). When the percentages of monocyte subpopulations in the presence or absence of TNF were determined, we observed no alterations in the migration of classical (Fig. 6l) or intermediate (Fig. 6m) monocytes and a decrease in the migration of nonclassical monocytes (Fig. 6n) toward IOs. Interestingly, we observed a significant decrease in classical (Fig. 6l,o) monocyte migration and an increase in intermediate and nonclassical (Fig. 6m–o) monocyte migration in TNF + serotonin-pretreated IOs compared with TNF-pretreated IOs. We also observed an increase in intermediate monocyte migration (Fig. 6m) and a decrease in nonclassical (Fig. 6n) monocyte migration toward the intestinal tissue when the IOs were pretreated with serotonin alone. Considering the differences in the migration of the different populations of monocytes, we focused on the migratory and activation markers of the cells. Interestingly, we did not observe any significant differences in CD11b expression on monocytes when TNF-pretreated and TNF + serotonin-pretreated samples were compared (Fig. 6p). Conversely, we observed a significant decrease in CD36, CD86 and HLA-DR expression on monocytes that migrated toward IOs pretreated with TNF + serotonin compared with those cultured with IOs treated with TNF alone (Fig. 6q). Overall, the evidence implies that serotonin affects healthy and inflamed intestinal tissue, resulting in alterations in the attraction of monocytes toward the tissue.

## Discussion

The roles of neuromodulators in gut homeostasis and the different mechanisms involved have been comprehensively described^37^. However, the mechanistic interconnections between neuromodulatory signals in the human gut mucosa and the immune system are not well known. As the intestinal tissue is the main source of serotonin, we hypothesized that serotonin might affect the immune response, the migration of immune cells and homeostasis in the mucosal tissue. Using state-of-the-art 3D models, we aimed to create a human cell-based model of TNF-induced inflammation and thus overcome the limitations of animal and two-dimensional cell line models. Here, we aimed to study the effects of neuromodulators, especially serotonin, on the mucosal tissue and resident immune cells and their roles in controlling inflammation and resolving controversies in mouse and rat models, revealing their opposing effects.

Here, we showed that IOs exhibit distinct expression patterns of TLRs, with TLR2 and TLR6 being expressed mostly by epithelial cells, TLR3 being expressed mainly in mesenchymal cells and TLR4 being expressed in both epithelial and mesenchymal cells. So far, only the mouse intestine TLR expression map is available^38^. Interestingly, compared with this map, we observed different TLR localizations within the IOs, pinpointing interspecies differences between mouse and human intestinal tissues. The influence of the microbiota on intestinal neural tissue and its production of neuromodulators has been intensively studied and reviewed^39–41^. However, how neuromodulators affect the expression of TLRs and thus the sensing of the microbiota is not known. In this study, we showed that stimulation of IOs with neuromodulators, namely dopamine, noradrenaline, acetylcholine and serotonin, induced a decrease in the expression of various TLRs, including TLR2 and TLR4. Interestingly, we observed different effects of neuromodulator stimulation on the mRNA and protein expression levels of TLRs in IOs, suggesting the direct regulation of innate immune signaling by these neuromodulators. In this study, we observed a significant decrease in the protein expression of TLR4 and TLR6 upon stimulation with all the studied neuromodulators, including serotonin, dopamine, acetylcholine and noradrenaline. However, in contrast to serotonin and dopamine, acetylcholine and noradrenaline did not induce a decrease in the protein expression of TLR2 and TLR3 in IOs, highlighting their important and hence distinct roles in the mucosal tissue and the immune response. Our results also suggest that the decreased dopamine and serotonin levels observed in inflamed tissue from patients with IBD^42,43^ might be linked to altered expression of TLRs in these patients. The quantification of RNA and protein expression in intestinal tissue from cohorts of both pediatric and adult patients with IBD revealed significant increases in TLR2 and TLR4 expression in patient groups compared with healthy controls^44–46^. Approximately 50% of whole-body dopamine is produced in the gut^2–5^. In addition to its role in the central nervous system by controlling physiological functions such as hormone secretion or food intake, dopamine has also been implicated in gastrointestinal functions and has been suggested to be protective by maintaining intestinal homeostasis^2^. However, further research is needed to provide more insights into the effects of neuromodulators on the expression and signaling of TLRs in different cell types within human mucosal tissue.

Previously, we showed that iPS cell-derived organoids do not consist of any immune cells^32^ and thus enable the study of specific mechanisms of action of proinflammatory cytokines in mucosal tissue, which is challenging with conventional in vivo and ex vivo models. We focused on TNF and its effect on the intestinal tissue using our organoid model. During inflammation, TNF is mostly produced by immune cells, with intestinal tissue-resident MΦs and tissue-migrating monocytes being the major sources^47^. However, the response of the intestinal tissue to TNF and the subsequent effects on the immune response are still poorly understood. A pilot study in which mouse jejunal organoids were stimulated with TNF documented the induced expression of CXCL2 (MIP-2), emphasizing the important role of the intestinal tissue in the response to proinflammatory stimuli^48^. In accordance with these findings, our study demonstrated that, upon TNF stimulation, the mucosal tissue represents a crucial source of various chemokines and, therefore, plays a pivotal role in controlling the inflammatory response through the chemoattraction of immune cells. After TNF treatment, the expression of other chemokines important for the chemotaxis of innate and adaptive immune cells, including CXCL8 (IL-8), CCL2 (MCP-1), CCL11 (eotaxin-1) and CCL20, increased. A previous study revealed that short-term stimulation with TNF increased CXCL2 levels and decreased the mRNA expression of CGA^48^, a neuroendocrine secretory protein produced by EECs. However, in our dataset, we did not observe a significant alteration in CGA expression after stimulation with TNF, highlighting the time-dependent effect of TNF and more complex interactions with EECs. EECs produce a large variety of hormones and neuromodulators, including serotonin^49^, and are involved in chronic inflammation of the intestine^50,51^ and neurodegenerative disorders^39^, suggesting the close interconnectivity between TNF and serotonin in intestinal inflammation.

Previous studies have suggested a proinflammatory role for serotonin based on evidence obtained from mouse models of intestinal inflammation, where in an IL-10 knockout model, depletion of the serotonin transporter (SERT) significantly worsens general health and intestinal inflammation^28^. On the other hand, in a dextran sulfate sodium-induced colitis mouse model, a reduction in serotonin via the depletion of the tryptophan hydroxylase 1 (THP1) gene decreases the severity of colitis, which is associated with a decrease in MΦ infiltration and significantly decreased concentrations of proinflammatory cytokines such as TNF^29^. In another in vivo study, the depletion of SERT in mice was shown to affect the size of the intestinal villi and enterocyte cell division, confirming the importance of serotonin as a physiological regulator of intestinal growth under homeostatic conditions^52^. In contrast to reports based on mouse models, our results indicate that serotonin does not induce a proinflammatory response in human mucosal tissue. In an in vitro rat model, the activation of one of the serotonin receptors on smooth muscle cells suppressed multiple responses to TNF stimulation^30,36^. The authors showed that an agonist of the 5-HT2A receptor attenuated the TNF-induced proinflammatory response by decreasing the expression of IL-6, ICAM–1 and VCAM-1^30^. In our study, we obtained similar findings using an in vitro human model, and our results indicated the distinct effect of serotonin on TNF-induced inflammation, revealing that serotonin and TNF signaling synergize to interfere with human gut pathophysiology. Our findings suggest that the results obtained in mouse models need to be interpreted cautiously when translated to human health research. Importantly, this study suggests that rat models are probably more accurate than in vivo models in this context. Our study focused on the clinical relevance and effects of serotonin on healthy and inflamed intestinal tissues, and the findings indicate that further research focusing on the molecular pathways involved in the interactions between serotonin and TNF signaling is needed.

The excess infiltration of immune cells in the gut tissue drives many pathologies and even exacerbates existing inflammation. Monocytes, in particular, are pluripotent plastic cells that can differentiate and perform various effector functions after migrating to target tissues^12^, thus influencing the course and resolution of inflammation in various human pathologies. A recent study using single-cell spatial transcriptomics revealed the presence of inflammation-dependent alternative (IDA) MΦs, specifically in the colon of patients with IBD^53^. IDA MΦs expressed neuregulin 1, a molecule involved in epithelial cell expansion and differentiation, suggesting their role in epithelial regeneration during inflammation. Interestingly, the authors reported that IDA MΦs were transcriptionally similar to MΦs derived from monocytes stimulated with macrophage colony-stimulating factor (M-CSF) and serotonin^53^. These results emphasize the crucial role of the tissue microenvironment in monocyte/MΦ differentiation. Our study provides the first evidence that serotonin can alter mucosal tissue pathology by promoting the migration of intermediate and nonclassical monocytes during TNF-induced inflammation without affecting the chemotactic ability of monocytes in healthy tissue. In particular, serotonin seems to upregulate the expression of genes involved in pathways such as cell–cell adhesion. In contrast, in TNF-induced inflammation, serotonin exerts a pleiotropic effect on monocyte migration by altering both tissue chemokine expression and homeostasis to favor the migration of nonclassical monocytes. Compared with classical monocytes, nonclassical monocytes have distinct transcriptomic profiles and functions^54^. In addition to their antigen-processing capabilities, they differ from classical monocytes in terms of their metabolic profiles and associations with wound-healing processes^55^. Interestingly, the expression of CD11b, a molecule important for the adhesion and migration of monocytes and MΦs^56^, was not affected in monocytes that migrated toward TNF + serotonin-treated organoids. These findings suggest that the altered migration of distinct monocyte subsets is caused by different mechanisms. In contrast, we observed a decrease in CD36, CD86 and HLA-DR expression on monocytes migrating toward the tissue stimulated with TNF in the presence of serotonin. CD36 is a scavenger receptor involved in many immune cell functions, such as phagocytosis and monocyte activation^57^, whereas CD86 is a costimulatory molecule that is important for the proper activation of lymphocytes and thus plays a critical role in the adaptive immune response^58–60^. Overall, our findings imply that serotonin can alter the profiles of immune cells and their migratory potential, even without direct contact with the cells. These findings indicate that serotonin influences the interplay between the intestinal tissue and both the innate and adaptive immune systems to regulate inflammation. The important role of neuromodulators in the intestinal immune response is further underscored by findings showing the effects of noradrenaline on dendritic cell migration, cytokine production and T cell polarization^37,61–64^. In contrast, in our study, we did not observe an effect of serotonin on the chemotaxis of either cytotoxic (CD8^+^) or helper (CD4^+^) T cells, underscoring the specific role of serotonin in modulating monocyte and tissue interactions rather broadly affecting the adaptive immune response. Furthermore, our results and those of previous studies have shown that neuromodulators play crucial roles in the mucosal immune response, with distinct roles and effects on innate and adaptive immunity.

Here, we describe the first evidence for the impact of serotonin on the human gut tissue obtained using human 3D iPS cell-derived IOs. Collectively, our findings reveal a new role for serotonin as a neurotransmitter that mitigates TNF-induced inflammation in human gut-like tissue and provide novel mechanistic insights into both its direct effect on the gut and its indirect effect on the immune system in contact with the gut tissue. We showed that serotonin modulates the mucosal tissue to alter the migratory potential of monocytes and change the phenotypes of the migrating cells. Therefore, serotonin seems to be the link connecting the nervous system to the intestinal tissue and monocyte/MΦ plasticity. Thus, it contributes not only to the bidirectional gut‒brain axis, but also to a signal involved in three-way crosstalk between the gut, the immune system and the brain. Nevertheless, further research is needed to elucidate the molecular mechanisms underlying the effects of serotonin observed in our study with a human cell-based model. These findings can shed light on the serotonin signaling pathway as a new potential therapeutic target for patients with IBD. Despite advances in the management of IBD, many patients remain nonresponsive to treatment; therefore, the development of novel therapeutic strategies is still needed. SERT inhibitors are widely used as antidepressant drugs to treat anxiety and depression in patients with IBD; furthermore, evidence of their effects on IBD-related complications is emerging^65^. Intriguingly, a recent study revealed that the use of antidepressants, including serotonin agonists and SERT inhibitors, was associated with a reduced risk of surgery in patients with IBD^66^. Our results highlight critical differences in mouse, rat and human physiology, and we showed that hiPS cell-derived organoids serve as a relevant model for future translational research focused on the effects of serotonin agonists and SERT inhibitors on alleviating intestinal inflammation in patients with IBD.

In addition to IBD, further research on the role of serotonin in inflammation could expand to investigations of other chronic inflammatory conditions, autoimmune disorders or neuroinflammatory diseases such as multiple sclerosis or Alzheimer’s disease, where the involvement of immune cells, including microglia, may be influenced by serotonin signaling. In fact, the effects of serotonin on microglial function and neuroplasticity have been extensively studied, revealing the pleiotropic role of serotonin in microglial biology^67,68^. Although we did not observe altered chemotaxis of either T cells or B cells upon serotonin stimulation of IOs, further research could focus on the interaction of these cells with the mucosal tissue or cytokine and the effects of serotonin on chemokine production to better understand its role in the adaptive immune response. Additionally, exploring how serotonin receptor subtypes modulate specific immune functions and migration in different inflammatory settings could identify novel therapeutic targets for treating autoimmune disease, neuroinflammation and chronic inflammatory conditions beyond IBD.

## Supplementary information

Supplementary Information

## Data Availability

The data supporting the findings of this study are available within the paper and its Supplementary Information or can be obtained from the corresponding authors upon reasonable request. The datasets generated during the current study are available from GEO (accession no. GSE269175).
