# Supplementary material for: Serotonin attenuates tumor necrosis factor-induced intestinal inflammation by interacting with human mucosal tissue
Source: Exp Mol Med. 2025 Feb 3;57(2):364–78. doi: 10.1038/s12276-025-01397-1 (PMC11873120; doi:10.1038/s12276-025-01397-1)
Supplement: Supplementary file 1 — Supplementary Information [file 12276_2025_1397_MOESM1_ESM.pdf]

SUPPLEMENTARY INFORMATION

Serotonin attenuates tumor necrosis factor alpha-induced intestinal inflammation by interacting with human mucosal tissue

Veronika Bosáková, Ioanna Papatheodorou, Filip Kafka, Zuzana Tomášiková, Petros Kolovos, Marcela Hortová Kohoutková, Jan Frič

This Supplementary information contains Supplementary Table 1 and Table 2, showing details of used antibodies and TaqMan^{TM} probes.

Supplementary table 1. Details of used antibodies

Antibody	Conjugate	Clone	Dilution	Vendor	Host
**CD11b**	BV510	ICRF44	1:100	BioLegend	Mouse
**CD14**	PE	63D3	1:100	BioLegend	Mouse
**CD16**	BV711	3G8	1:100	SONY	Mouse
**CD16**	eFluor 450	eBioCB16	1:100	eBioscience	Mouse
**CD19**	Biotin	HUB19	1:100	eBioscience	Mouse
**CD3**	BV650	UCHT1	1:100	BD Horizon	Mouse
**CD36**	PE-Cy7	5-271	1:100	BioLegend	Mouse
**CD4**	BV510	SK3	1:100	BioLegend	Mouse
**CD45**	BV421	2D1	1:100	BioLegend	Mouse
**CD45**	PE-Cy7	2D1	1:100	BioLegend	Mouse
**CD8**	BV421	RPA-T8	1:100	BioLegend	Mouse
**CD86**	BV711	IT2.2	1:100	BioLegend	Mouse
**CD90**	-	Polyclonal	1:100	R&D	Sheep
**CD90**	PerCP-Cy5.5	eBio5E10	1:100	eBioscience	Mouse
**CGA**	-	LK2H10	1:100	Invitrogen	Mouse
**E-cadherin**	-	24E10	1:300	Cell Signaling TECHNOLOGY	Rabbit
**E-cadherin**	Alexa Fluor 488	DECMA-1	1:300	eBioscience	Rat
**EpCam(CD326)**	Biotin	1B7	1:200	eBioscience	Mouse
**HLA-DR**	PE-Dazzle 594	L243	1:100	BioLegend	Mouse
**Lysozym**	-	SB1	1:100	Invitrogen	Mouse
**MUC5AC**	-	45M1	1:500	Abcam	Mouse

**Secondary anti mouse**	Alexa Fluor 546	Polyclonal	1:500	Invitrogen	Goat
**Secondary anti mouse**	Alexa Fluor 647	Polyclonal	1:500	Invitrogen	Donkey
**Secondary anti mouse**	Biotin	Polyclonal	1:500	Jackson Immuno Research	Goat
**Secondary anti rabbit**	Alexa Fluor 488	Polyclonal	1:500	Invitrogen	Donkey
**Secondary anti rabbit**	Alexa Fluor 555	Polyclonal	1:500	Invitrogen	Donkey
**Secondary anti Rat**	Alexa Fluor 488	Polyclonal	1:500	Invitrogen	Donkey
**Secondary anti sheep**	Alexa Fluor 546	Polyclonal	1:500	Invitrogen	Donkey
**Streptavidin**	Alexa Fluor 514	-	1:500	Invitrogen	-
**Streptavidin**	APC	-	1:500	BioLegend	-
**TLR2**	-	D7G9Z	1:200	Cell Signaling TECHNOLOGY	Rabbit
**TLR3**	PE	TLR3.7	1:200	eBioscience	Mouse
**TLR4**	-	76B357.1	1:200	Invitrogen	Mouse
**TLR6**	-	Polyclonal	1:100	Sigma	Rabbit

Supplementary table 2. Details of used TaqMan™ probes

Gene ID	TaqMan Probe ID
**TLR2**	Hs00610101_m1
**TLR3**	Hs01551078_m1
**TLR4**	Hs00152939_m1
**TLR5**	Hs01920773_s1
**TLR6**	Hs01039989_s1
**TLR7**	Hs00152971_m1
**TLR8**	Hs07292888_s1
**TLR9**	Hs00370913_s1
